# Altered Gene Expression Encoding Cytochines, Grow Factors and Cell Cycle Regulators in the Endometrium of Women with Chronic Endometritis

**DOI:** 10.3390/diagnostics11030471

**Published:** 2021-03-08

**Authors:** Ettore Cicinelli, Amerigo Vitagliano, Vera Loizzi, Dominique De Ziegler, Margherita Fanelli, Stefano Bettocchi, Claudia Nardelli, Giuseppe Trojano, Rossana Cicinelli, Crescenzio Francesco Minervini, Daniela Leronni, Luigi Viggiano

**Affiliations:** 1 2nd Unit of Obstetrics and Gynecology, Department of Biomedical and Human Oncologic Science, Policlinico University of Bari, 70124 Bari, Italy; ettore.cicinelli@uniba.it (E.C.); vera-loizzi@uniba.it (V.L.); stefano.bettocchi@uniba.it (S.B.); nardellicla@libero.it (C.N.); giutrojano@gmail.com (G.T.); cicinellirossana@gmail.com (R.C.); 2 Department of Women and Children’s Health, University of Padua, 35128 Padua, Italy; 3 Department of Ob Gyn and Reproductive Medicine, Foch Hospital, 92150 Suresnes, France; ddeziegler@me.com; 4 Interdisciplinary Department of Medicine, University of Bari, 70124 Bari, Italy; margherita.fanelli@uniba.it; 5 Department of Emergency and Organ Transplantation (D.E.T.O.), Hematology Section, University of Bari, 70124 Bari, Italy; eziominervini@gmail.com; 6 Neuroapoptosis Laboratory, Department of Neurological Surgery, University of Pittsburgh, Pittsburgh, PA 15213, USA; dleronni@gmail.com; 7 Department of Biology, University of Bari, 700124 Bari, Italy; luigi.viggiano@uniba.it

**Keywords:** endometrium, chronic endometritis, real time RT-PCR, gene expression, growth factors

## Abstract

To evaluate the expression of genes encoding cytokines, grow factors and cell cycle regulators in the proliferative endometrium of women with chronic endometritis (CE) compared to controls. We performed a case-control study on seven women with CE as diagnosed by hysteroscopy and histology (Cases) compared to six women without CE (Controls). All women underwent diagnostic hysteroscopy plus endometrial biopsy during the mid-proliferative phase of the menstrual cycle. Endometrial samples were divided into two different aliquots for histological and molecular analyses. The endometrial expression profile of 16 genes encoding proteins involved in the inflammatory process, proliferation and cell cycle regulation/apoptosis was assessed by using high-throughput qPCR. Study endpoints were between-group differences in the expression of VEGF A, VEGF B, VEGF C, EGF, TNF, TGF B1, IFNG, TP73, TP73L, BAXva, CDC2, CDC2va, CCND3, CCNB1, BAX and IL12. RESULTS: VEGF A, VEGF B, VEGF C, EGF, TNF, TGF B1, IFNG, TP73, TP73L, BAXva, CDC2, CDC2va, CCND3, CCNB1 were significantly overexpressed in women with CE compared to controls, while BAX and IL12 had similar expression between groups. In women with CE, we found an altered endometrial expression of genes involved in inflammatory, cell proliferation, and apoptosis processes. The dominance of proliferative and anti-apoptotic activity in CE may potentially promote the development of polyps and hyperplastic lesions.

## 1. Introduction

In the last years, a growing interest has focused on chronic endometritis (CE), defined as the chronic inflammation of endometrial mucosa [1,2]. CE is a subtle pathology accompanied by mild and unspecific disturbances. Importantly, CE may interfere with reproductive capacity, mostly by altering endometrial receptivity [3,4,5,6,7,8,9].

Diagnosing CE is challenging. CE may be diagnosed by fluid hysteroscopy [2] but the accuracy of this technique is strongly dependent on operator’s expertise. Recently, an expert panel developed a series of hysteroscopic diagnostic criteria with the purpose of simplifying CE recognition, with encouraging results. Nevertheless, these criteria still need a clinical validation though prospective studies [10]. Currently, histology remains the gold standard for the diagnosis of CE, based on the identification of plasma cells in the endometrial stroma by CD138 immunostaining [8,9]. 

Histologically, CE is characterized by both quantitative and qualitative alteration in leukocyte infiltration. A large number of B cells infiltrate both the endometrial functional layer and the basal layer, trespassing on the glandular epithelial areas and invading further into the gland lumina. Moreover, a lower percentage of CD16 negative CD56 positive-bright natural killer cells and an increase in T cells were found in the secretory phase of women with CE [10,11]. 

Endometrial inflammation and leukocyte infiltration cause an altered local biochemical and paracrine production in CE, which results in abnormal endometrial function, menstrual disorders and impaired receptivity to the embryo. Moreover, the persistence of chronic endometrial inflammation has been associated with the onset of endometrial proliferative pathologies like endometrial polyps [12] and, virtually, to carcinogenetic processes [13]. 

In a previous study on women with CE during the mid-secretory phase (day 20–22 of the cycle), we found an altered expression of genes encoding some proteins involved in endometrial inflammation, replication and apoptosis processes [14]. These alterations may presumably exert a negative effect on endometrial receptivity to the embryo, therefore justifying the association between CE and impaired reproductive function. 

Notably, during the mid-secretory phase, endometrial gene expression is physiologically modified compared to the proliferative phase. A number of substances and factors are released in the uterine cavity with the purpose of promoting embryo-uterine crosstalk. For this reason, basing on a single study on mid-secretory endometria, it cannot be assumed that endometrial gene expression is altered throughout the endometrial cycle in CE.

Over that background, in the present study we aimed to evaluate the expression of genes involved in inflammation, cell replication and apoptosis in the proliferative endometrium of women with CE during. Specifically, we analyzed 16 genes encoding proteins involved in the inflammatory response (IL12 (interleukin-12), INFG (interferon-ℽ)), proliferation (TGFB1 (transforming growth factor β-1), TNF (tumor necrosis factor), VEGFA (vascular endothelial growth factor-A), VEGFB (vascular endothelial growth factor-B), VEGFC (vascular endothelial growth factor-C), EGF (epidermal growth factor)), cycle cell regulation CDC2 (cell division control protein 2), CDC2va (cell division control protein variant), CCND3 (cyclin D3), CCNB1 (cyclin B1) and apoptosis (TP73 (tumor protein P73), TP63 (Tumor protein P63), BAX (BCL-2 associated X protein) transcript variant alpha, BAX transcript variant beta). 

## 2. Materials and Methods

### 2.1. Study Design

This was a case-control study realized at Department of Ob/Gyn, University of Bari, Italy. We enrolled consecutive patients referred for diagnostic hysteroscopy due to abnormal uterine bleeding or reproductive issues between January–August 2019. 

The study was approved by our Institutional Review Board (IRB protocol number 5689/2018, date 9 February 2018) and written informed consent was obtained from each study participant.

### 2.2. Participants and Interventions

We enrolled a total number of 13 participants. Seven women were diagnosed with CE at fluid hysteroscopy and histology (cases). Controls were a group of six women undergone hysteroscopy plus biopsy in which CE diagnosis was excluded. Hysteroscopy was performed during the proliferative phase (i.e., day 6 to 10) of the cycle. After diagnostic hysteroscopy, all the patients underwent endometrial biopsy by using a curette. Endometrial samples were divided into two different aliquots for histological and molecular analyses. The samples for molecular analysis were immediately immersed in liquid nitrogen to avoid RNA degradation. All details about inclusion criteria, hysteroscopic examination, endometrial sampling and histological analysis are reported in Supplementary File.

### 2.3. Gene Expression Profiling

Total RNA was isolated using Trizol Reagent (Invitrogen S.R.L, Milan, Italy) according to our published protocol (14). Gene expression level was determined by quantitative polymerase chain reaction (qPCR). The cDNA was obtained using the QuantiTect Reverse Transcription Kit (QIAGEN, Milan, Italy) according to handbook protocol. The cDNA was amplified using the Applied Biosystems Real-Time PCR System 7300 in the presence of SYBR Green I. The optimization of real-time PCR reaction was performed according to the manufacturer’s instructions (Applied Biosystems, Foster City, CA, USA).

The PCR conditions were as follows: 2 min at 50 °C, 10 min at 95 °C, followed by 40 cycles of 15 s at 95 °C and 1 min at 60 °C for all the primer pairs used. At the end of each reaction, the cycle threshold (Ct) was manually set up at a level that reflects the best kinetic PCR parameters. Melting curves were acquired and analyzed to control for specificity.

Validated QuantiTect Primer (Qiagen) were used for the following genes: VEGFA; VEGFB; VEGFC; EGF; TNF; TGFB1; IFNG; IL12A; TP73; TP73L; BAX; BAXva; CDC2; CDC2va; CCND3; CCNB1.

The level of gene expression was calculated using the relative quantification approach based on the DDCt method. We used the 28S rRNA as reference gene and a cDNA pool of 5 normal donors as golden standard/calibrator. The efficiencies of the target and reference genes were similar. Gene expression comparisons between CE and control groups were performed by U-Mann Whitney test. The analysis of data was performed by SPSS version 23 (Chicago, IL, USA).

## 3. Results

**Figure 1:**
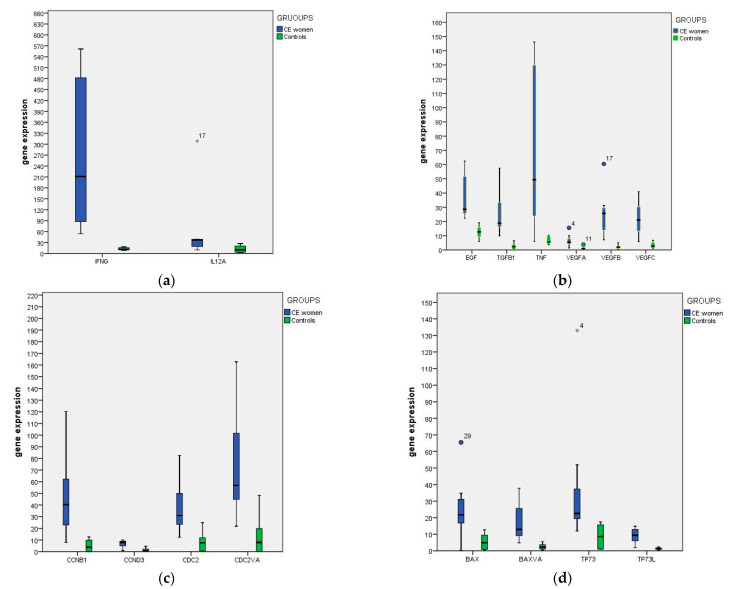
Distribution of relative quantification by real-time qPCR of selected genes expression in patients (blue box) respect to control females (green box). The genes were grouped according to their function: inflammatory response (panel **a**), proliferation (panel **b**), cell cycle regulation (panel **c**) and apoptosis (panel **d**). Significant statistical results are marked (* *p* < 0.05).

The baseline characteristics of patients were similar between groups (Table 1). Endometrial gene expression profile showed significant differences in women with CE compared to controls (Table 2) (Figure 1a–d).

Among the genes related to inflammatory response (Figure 1, panel a), INFG showed higher levels in CE compared to controls (*p* < 0.05). Moreover, all genes related to proliferation showed higher levels in CE compared to controls. (*p* < 0.05; Figure 1, panel b). Similarly, all the genes related to cell cycle regulation (Figure 1, panel c) were significantly higher in CE women compared to controls (*p* < 0.05). Regarding apoptosis function (Figure 1, panel d), all the genes but not BAX showed higher expression in CE women compared to controls (*p* < 0.05).

## 4. Discussion

Our study first demonstrates that the proliferative endometrium of patients with CE displays an altered expression of genes involved in the inflammatory response, cell proliferation and apoptosis. These data are complementary to those of our previous study on gene expression analysis during the mid-luteal phase in women with CE [14]. As the sum of our experiences, we can assume that the endometrium of women with CE suffers from a sustained alteration of anti- to pro-apoptotic factors ratio that is independent from the stage of the menstrual cycle. This assumption may partly explain different issues associated with CE, including reproductive impairment, abnormal uterine bleeding and the development of proliferative lesions such as micro- and macro-polyps [12,15,16].

**Figure 2:**
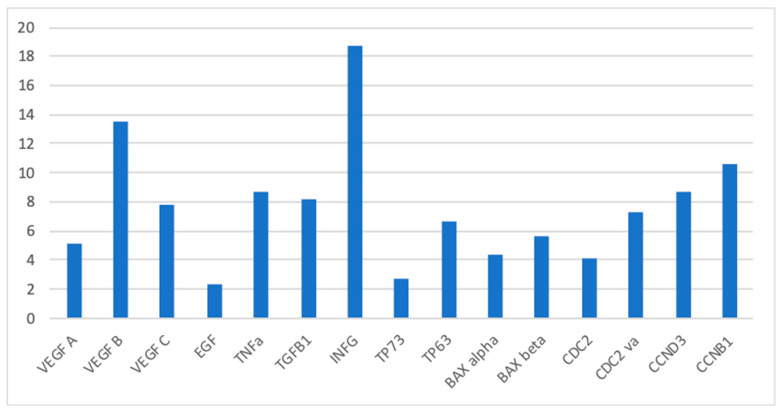
The ratio between the median value in CE and control groups is displayed.

In this study, we found that the expression profile of VEGFA, VEGFB, VEGFC, EGF, TNF, TGFB1, IFNG, TP73, TP63, BAXva, CDC2, CDC2va, CCND3, CCNB1 in the endometrium of women with CE was significantly different from controls (Figure 1a–d). The ratio between the median value in CE and control groups is displayed in Figure 2.

VEGF (vascular endothelial growth factor) is a sub-family of growth factors playing an important role in neo-vascularization by activating tyrosine kinase receptors [17]. VEGFA is a key regulator of angiogenesis, promoting endothelial cell migration and proliferation [18]. Moreover, through endothelial nitric oxide synthase regulation, it can increase vascular permeability and vasodilatation [19], whereas increased Akt signaling gives VEGFA a role in cell-survival control [20]. VEGFB is expressed in many types of cells and its major role lies in safeguarding tissues and cells from oxidative stress-induced damage [21]. On the other hand, over-expression of VEGFC results in selective induction of lymphatic proliferation [17].

In the human endometrium, VEGF expression is cycle-dependent and modulated by ovarian steroids [22]. In women diagnosed with CE, we found a significant increase in RNAs expression of all the VEGF isoforms investigated. Interestingly, VEGF B was more expressed than isoforms A and C (with a median ratio of 13.5 compared to 5.2 and 7.8, respectively). We can speculate that the up-regulation of VEGF A and C may induce neo-lymphoangiogenesis and endometrial proliferation; the sharp increase in VEGF B may represent a tentative in safeguarding cells by oxidative stress caused by inflammation.

Moreover, EGF (epidermal growth factors) showed higher expression in women with CE compared to controls (with a median ratio of 2.4). EGF is the prototype of a family of ligands produced as transmembrane precursors shed from the cell surface by proteolytic processing by metalloproteases. The mature soluble factors are involved in autocrine and paracrine regulations via binding and activation of specific tyrosine kinase receptors (EGFR/ErbB1, ErbB2–4) [23,24,25]. EGF binding to EGFR results in cellular proliferation, differentiation and survival. Consistently cell cycle regulatory factor Cyclin D1 was also upregulated upon EGF and HGF addition [26].

Furthermore, TNFα (tumor necrosis factor) showed a marked increase in women with CE compared to controls, with a median ratio of 8.7. TNFα is a cell signaling protein (cytokine) involved in systemic inflammation and is one of the leading cytokines of the acute phase reaction. It is produced mainly by activated macrophages, although it can be produced by other cell types. TNFα is a multifunctional cytokine involved in cell survival, proliferation, differentiation and death. Dysregulation of TNF production has been implicated in a variety of human diseases including cancer and inflammatory bowel disease [27]. In our experience, TNFα was up-regulated in CE. This finding was in agreement with a previous study in which we found elevated levels of TNF in menstrual effluent of women with CE [28]. Exposure to TNFα rises estrogen biosynthesis in endometrial glandular cells, which may be associated with occurrence of endometrial micropolyposis, namely a typical hysteroscopic sign of CE [29].

TGF-β1 (transforming growth factor-beta 1) expression was raised by 8.2 fold in women with CE. TGF-β1 is a secreted protein involved in many cellular functions, including the control of cell growth, cell proliferation, cell differentiation, and apoptosis. TGF-β1 is expressed in human endometrium and regulates epithelial cell proliferation and apoptosis, besides exerting an autocrine pro-apoptotic effect on human endometrial stroma, via the FasL/Fas system [30,31,32]. Notably, in a recent paper, Wang and co-workers found that in women with CE complaining of recurrent implantation failure (RIF) the TGF-β1 were significantly lower than controls [33]. We can only speculate that different populations (RIF patients vs. mostly abnormal uterine bleeding (AUB) patients) could play a role in explaining contrasting results. However, TGF-b and IL-6 stimulated naive T cells to induce a Th17 response with stimulation of inflammatory and immunitary response. So, high levels of TGF-β1 may indicate a derangement in the delicate balance between pro- and anti-inflammatory activity played by TGF-β1 at the endometrium level. IFN-γ (interferon gamma) is pleiotropic cytokine with antibacterial, antiproliferative and immunomodulatory effects on numerous target cells. IFN-γ is mainly produced by activating T lymphocytes and by NK cells. IFN-γ synergizes with LPS-induced production of IL-1, IL-6, and TNFA in macrophages. It was 18, 75 fold higher in CE compared to controls, potentially suggesting a local alteration in the immune response. IFN-γ is produced predominantly by natural killer (NK) and natural killer T (NKT) cells as part of the innate immune response, and by CD4 Th1 and CD8 cytotoxic T lymphocyte (CTL) effector T cells once antigen-specific immunity develops [34].

IL12 in women with CE were 3.6 higher compared to controls but the difference was not significant. IL-12 is a heterodimeric cytokine produced by phagocytic cells and professional antigen-presenting cells. IL 12 production is induced by bacteria, intracellular pathogens, fungi and viruses. It acts on T and natural killer cells, and has a broad array of biological activities. This cytokine is required for the T-cell-dependent induction of interferon-gamma (INF-γ) and is important for the differentiation of both Th1 and Th2 cells. It as antitumor effect in the initial phase of CE but then it also appears to support neoplastic transformation.

As regard as TP73 (tumor protein 73) and TP63 (tumor protein 63), they raised by 2.7 and 6.7 times in CE, respectively. TP73 encodes a member of the p53 family transcription factors involved in cellular responses to stress and development promoting apoptosis in response to DNA damage [35,36]. Analyses of many tumors typically found in humans (including breast, endometrial and ovarian cancer) show an over-expression of p73 [37].

The BAX gene is a member of the Bcl2 family that has a pro-apoptotic function to regulate the programmed cell death process [34]. A number of BAX isoforms have been previously identified: alpha, beta, gamma, delta and omega. We found that in women with CE median BAX alpha gene expression was 4.4 fold change higher than controls (not statistically significant) while BAX variant beta gene median levels were 5.6 fold change higher than controls. The slight BAX alpha variant gene activation with pro-apoptotic effect may explain the prevalence of proliferative and anti-apoptotic effects in case of CE with abnormal proliferative effect on the endometrium, resulting in the formation of polyps [14].

Finally, we analyzed the expression of cyclins, which act as regulators of CDK kinases. Down-regulation of cyclins has been linked to a number of malignant neoplasms including endometrial hyperplasia and endometrial cancer [38,39,40,41]. Compared to controls, the median CDC2, DCDC2va, CCND3 and CCNB1 gene expression in CE increased significantly (by 4.1 fold, 7.3 fold, 8.7 fold and 10.6 fold). CDC2 gene produces a protein Cyclin-dependent kinase 1 (CDK1) that functions as a serine/threonine kinase that is a key player in cell cycle regulation [40,41]. With its cyclin partners, CDK1 forms complexes that phosphorylate a variety of target substrates leading to cell cycle progression [37]. CCND3 (cyclin D3) activates the respective protein kinase CDK4 promoting G1/S phase transition. CCNB1 (cyclin B1) acts as an activator to cyclin-dependent kinase 1(CDK1) essential for G2/M phase transition. Notably, cyclins and CDKs are a critical factor in proliferation and differentiation of endometrial cells. [38,41]. Progesterone may inhibit cell proliferation, mediate G2/M cell cycle arrest and induce apoptosis in hECs via down-regulating Cyclin B1. During (endometrioid) endometrial carcinogenesis, there is increasing proliferation paralleled by the progressive derailment of cyclin B1, cyclin D1, cyclin E, p16, p21, p27, p53 and cdk2, indicating the importance of these cell cycle regulators in endometrial carcinogenesis [42].

### Study Strengths and Limitations

Originality and rigorous methodology are points of strength of our study. We demonstrated for the first time that morphological and histological alteration in CE correspond to altered endometrial paracrine environment. The small sample of patients included in the analysis certainly represent the main study limitation. Nevertheless, the huge differences between comparators in terms of gene expression may allow drawing reasonable conclusions from the data.

## 5. Conclusions

The proliferative endometrium of women affected by CE showed altered expression of different genes codifying pro-inflammatory cytokines and factors involved in growth and apoptosis processes. Specifically, we found an over-expression of cytokines, growth factors and cyclins favoring endometrial proliferation against apoptosis.

Our findings may support the etiopatogenetic role of CE in the development of different endometrial disorders including impaired receptivity to the embryo, abnormal bleeding and proliferative diseases such as polyps and hyperplastic lesions.

## Figures and Tables

**Table 1:** Main clinical characteristics of patients. Data are a range and median or number of cases (*n*). BMI: body mass index; AUB: abnormal uterine bleeding.

Variables	CE Group (*n* = 7)	Control Group (*n* = 6)	*p* Value
Age (years)	24–39, 30.5	27–35, 30	NS
BMI (kg/cm^2^)	24–35, 29.5	23–33, 29.5	NS
Parity (*n*)	1–2, 1	1–2, 1	NS
AUB (*n*)	3	1	NS
Smoking (*n*)	0	1	NS
Previous intrauterine instrumentation	0	0	NS

**Table 2:** Endometrial gene expression profile in chronic endometritis (CE) women and in controls. U-Mann Whitney test results. * Statistically significant.

		Endometrial Expression							
	Full Name	CE Women			Controls			Median Ratio (CE Women/Controls)	*p* Value
		Median	Min	Max	Median	Min	Max		
IL12A	INTERLEUKIN 12A	36.45	9.79	308.44	10.17	2.53	27.24	3.6	0.151
IFNG	INTERFERON GAMMA	211.32	53,91	561,42	11,27	8.56	18.52	18.75	0.001 *
TGFB1	TRANSFORMING GROWTH FATOR B	18.89	9.97	57.48	2.27	0.16	6.55	8.2	0.001 *
TNF	TUMOR NECROSIS FACTOR (TNF SUPERFAMILY, MEMBER 2)	49.4	5.9	146.05	5.67	3.76	10.27	8.7	0.015 *
VEGFA	VASCULAR ENDOTHELIAL GROWTH FACTOR A	5.53	1.46	15.54	1.05	0.72	3.83	5.2	0.008 *
VEGF B	VASCULAR ENDOTHELIAL GROWTH FACTOR B	25.71	7.14	60.44	1.98	0.2	5.1	13.5	0.001 *
VEGF C	VASCULAR ENDOTHELIAL GROWTH FACTOR C	21.13	5.8	40.92	2.71	0.85	6.79	7.8	0.005 *
EGF	EPIDERMAL GROWTH FACTOR	28.65	22.2	62.43	12.67	5.84	19.14	2.4	0.017 *
CDC2	CELL DIVISION CONTROL 2	30.94	12.32	82.55	7.49	0.09	24.77	4.1	0.005 *
CDC2 va	CELL DIVISION CONTROL 2 VARIANT	56.86	21.82	162.69	7.79	0.18	48.12	7.3	0.008 *
CCND3	CYCLIN D3	7.77	1.01	10	0.88	0.15	4.65	8.7	0.014 *
CCNB1	CYCLIN B1	40.24	7.91	120.24	3.78	0.06	12.6	10.6	0.005 *
TP73	TUMOR PROTEIN P73	22.55	11.96	132.95	8.53	0.94	17.35	2.64	0.005 *
TP73L	TUMOR PROTEIN P73	9.39	1.87	14.88	1.4	0.29	2.34	6.71	0.004 *
BAX	BCL2-ASSOCIATED X PROTEIN	21.73	0.22	65.51	4.87	0.28	12.69	4.46	0.051
BAX va	BCL2-ASSOCIATED X PROTEIN VARIANT	12.91	4.83	37.83	2.26	0.32	5.46	5.71	0.002 *

## Supplementary Materials

The following are available online at https://www.mdpi.com/2075-4418/11/3/471/s1, Supplementary file: all details about inclusion criteria, hysteroscopic examination, endometrial sampling and histological analysis are reported in Supplementary File.
