# Supplementary material for: Altered Gene Expression Encoding Cytochines, Grow Factors and Cell Cycle Regulators in the Endometrium of Women with Chronic Endometritis
Source: Diagnostics (Basel). 2021 Mar 8;11(3):471. doi: 10.3390/diagnostics11030471 (PMC7999985; doi:10.3390/diagnostics11030471)
Supplement: Supplementary file 1 [file diagnostics-11-00471-s001.pdf]

Inclusion criteria

We included only women with no abnormalities at both clinical examination and transvaginal ultrasound. All patients were free of any hormonal or antibiotic treatment for the last six months. Women with an abnormal uterine cavity (presence of submucosal fibroids or endometrial polyps), diabetes, thyroid diseases, hydrosalpinx, pelvic endometriosis, ovulation disorder, and polycystic ovarian syndrome were excluded from the study.

Diagnostic hysteroscopy and endometrial sampling

For hysteroscopy, a lens-based 2.9 mm hysteroscope, a 300 W light source with a xenon bulb and a 3 CCD (charge-coupled device) digital camera (Karl Storz, Tuttlingen, Germany) were used. Saline was employed to distend the uterine cavity at a pressure generated by the simple drop from a bag suspended 1 m above the patient. Diagnosis of CE was done based on hysteroscopic criteria previously published (2, 10). All hysteroscopies were performed by 3 out authors (G.T., V.P., C.N.) and records were revised and the diagnosis confirmed by 2 authors (E.C., A.V.) according to published criteria (10). Immediately after hysteroscopy, all women underwent endometrial biopsy using a 3 mm Novak's curette connected to a 20 ml syringe. Endometrial samples were divided into two different aliquots for histological and molecular analyses. The samples for molecular analysis were immediately immersed in liquid nitrogen to avoid RNA degradation.

Histological examination

Endometrial samples for histological examination were fixed in neutral formalin and embedded in paraffin according to routine histological procedure. Five microsections were stained with hematoxylin-eosin. A single operator (L.R.), who was unaware of the hysteroscopic findings, performed the histological examination following diagnostic criteria described in literature (2, 15). Superficial stromal edema, increased stromal density, pleomorphic stromal inflammatory infiltrate dominated by lymphocytes and plasma cells were considered as signs of inflammation. Subsequently,

endometrial tissue samples were examined by IHC (immunohistochemistry) using CD138 antibodies to detect for hematolymphoid subpopulations. The number of immunoreactive cells was identified under a high microscope magnification (400×) in 10 non-overlapping stromal areas. Chronic endometritis was defined as the presence of 1–5 plasma cells/hpf or discrete clusters of <20 plasma cells by CD138 staining.
